# WNT5B in Physiology and Disease

**DOI:** 10.3389/fcell.2021.667581

**Published:** 2021-05-04

**Authors:** Sarocha Suthon, Rachel S. Perkins, Vitezslav Bryja, Gustavo A. Miranda-Carboni, Susan A. Krum

**Affiliations:** ^1^Department of Orthopaedic Surgery and Biomedical Engineering, University of Tennessee Health Science Center, Memphis, TN, United States; ^2^Department of Experimental Biology, Faculty of Science, Masaryk University, Brno, Czechia; ^3^Department of Cytokinetics, Institute of Biophysics, Czech Academy of Sciences, Brno, Czechia; ^4^Division of Hematology and Oncology, Department of Medicine, University of Tennessee Health Science Center, Memphis, TN, United States; ^5^Center for Cancer Research, University of Tennessee Health Science Center, Memphis, TN, United States

**Keywords:** WNT5B, WNT signaling, development, cancer, WNT5A

## Abstract

WNT5B, a member of the WNT family of proteins that is closely related to WNT5A, is required for cell migration, cell proliferation, or cell differentiation in many cell types. WNT5B signals through the non-canonical β-catenin-independent signaling pathway and often functions as an antagonist of canonical WNT signaling. Although WNT5B has a high amino acid identity with WNT5A and is often assumed to have similar activities, WNT5B often exhibits unique expression patterns and functions. Here, we describe the distinct effects and mechanisms of WNT5B on development, bone, adipose tissue, cardiac tissue, the nervous system, the mammary gland, the lung and hematopoietic cells, compared to WNT5A. We also highlight aberrances in non-canonical WNT5B signaling contributing to diseases such as osteoarthritis, osteoporosis, obesity, type 2 diabetes mellitus, neuropathology, and chronic diseases associated with aging, as well as various cancers.

## Introduction

*Wingless-related integration site* (*Wnt*) genes are evolutionarily conserved, secreted proteins that are essential for developmental and biological processes. In 1982 the first *Int-1* gene was discovered and this proto-oncogene was activated by integration of the mouse mammary tumor virus to induce breast tumors (Nusse and Varmus, 1982), suggesting the importance of *Int* genes in cancer. The mutation of the *wingless* (*wg*) gene in Drosophila created the wingless phenotype (Sharma and Chopra, 1976) by interrupted segment polarity in fly larva (Rijsewijk et al., 1987), indicating its role in development. Over 30 years ago, the *wg* gene was shown to be a homolog with the mouse *Int-1* gene, leading to a combination of these two names to WNT (Rijsewijk et al., 1987).

The WNT family now contains 19 WNT genes, falling into 12 WNT subfamilies in mammalian genomes. All WNT genes encode proteins around 40 kDa in size and contain highly conserved cysteines (Miller, 2002; Clevers and Nusse, 2012). Mammalian WNT proteins are palmitoylated at conserved serine residues by a special palmitoyl transferase, Porcupine (PORCN), in the endoplasmic reticulum (Takada et al., 2006; Galli et al., 2007; Rios-Esteves et al., 2014). Zebrafish WNT3 is lipidated at both cysteine and serine residues (Dhasmana et al., 2021). The activity of PORCN is essential for the secretion of WNT ligands. Then, the seven-transmembrane protein Wntless/Evi (Wls) in the endoplasmic reticulum escorts mature hydrophobic WNT proteins to be secreted at the plasma membrane or released in exosomes, leading to both autocrine and paracrine effects (Banziger et al., 2006; Routledge and Scholpp, 2019).

**FIGURE 1:**
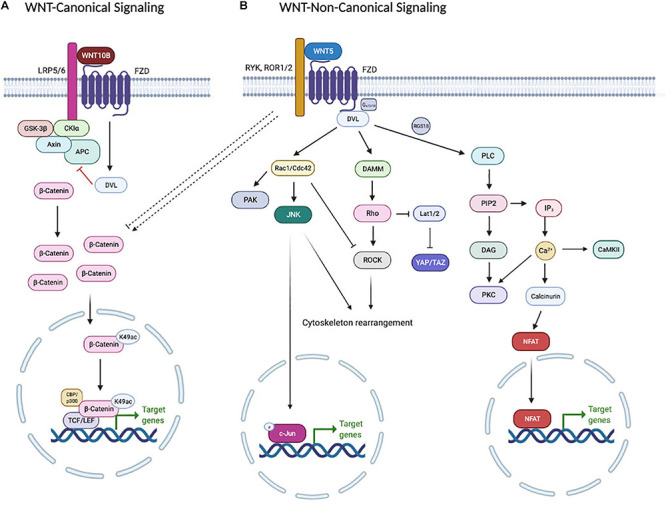
WNTs can signal through **(A)** β-catenin-dependent (or canonical) WNT signaling, **(B)** WNT-PCP signaling or WNT-Ca^2+^ signaling. See the text for details.

The WNT signaling pathway is divided into two main branches: the non-canonical (β-catenin-independent) signaling pathway and the canonical (β-catenin-dependent) signaling pathway (Figure 1). WNT ligands bind to various receptors and co-receptors. There are 10 members of the Frizzled (FZD) protein family, which serve as receptors for both the canonical and non-canonical signaling pathways. The pairing between specific FZDs and another receptor, such as low-density lipoprotein receptor-related protein 5 or 6 (LRP5/6), receptor Tyr kinase-like orphan receptor 1 or 2 (ROR1/2) and receptor Tyr kinase (RYK), directs the downstream signaling pathway (Azbazdar et al., 2021). Canonical WNT signaling is initiated by binding of WNT ligands (e.g., WNT3A and WNT10B) to a heterodimeric receptor complex formed by a Frizzled (FZD) and LRP5/6. The signaling output of the canonical WNT pathway is determined by the level of cytosolic β-catenin, which is under the strict control of the destruction complex. The destruction complex is composed of APC (Adenomatous Polyposis Coli), AXIN1, and two constitutively active kinases [glycogen synthase kinase (i.e., GSK3β) and casein kinase (i.e., CK1α)], which associate with β-catenin and promote its polyubiquitination by phosphorylating the degron motif of β-catenin (Stamos and Weis, 2013). Subsequently, phosphorylated β-catenin can be recognized by the F-box/WD-repeat protein β-TrCP within the SCF ubiquitin ligase complex, which facilitates the targeting of cytosolic β-catenin, leading to its proteasome-dependent degradation (Hart et al., 1999; Kitagawa et al., 1999). Binding of WNT ligands with FZD/LRP receptors induces stoichiometric sequestration of the destruction complex components AXIN1/CK1α/GSK3 onto the receptor complex and phosphorylation of LRP5/6, which are enhanced by Disheveled family members (DVL1-3). Receptor engagement leads to the accumulation of cytoplasmic β-catenin, which translocates into the nucleus, where it binds to members of the T cell factor/lymphoid enhancer factor (TCF/LEF) transcription factor family to drive transcription of WNT/β-catenin target genes such as *AXIN2* and *MYC* (amongst others) (Lecarpentier et al., 2019).

Some WNT ligands (e.g., WNT5A and WNT5B) can activate non-canonical WNT pathways, which are independent of β-catenin stabilization (Xiao et al., 2017). Non-canonical WNT signaling pathways are subdivided into the WNT-planar cell polarity (WNT-PCP) signaling pathway and the WNT-calcium (WNT-Ca^2+^) signaling pathway. Although these two pathways show overlapping receptors such as ROR and RYK, they utilize different downstream effectors (Chen et al., 2021). In the WNT-PCP signaling pathway, Disheveled (DVL) forms a complex with DVL-associated activator of morphogenesis 1 (DAMM1) to induce the small GTPase Ras homology family member (Rho) activation (Habas et al., 2001). Then Rho activates downstream kinases such as Rho-associated protein kinase (ROCK) (Winter et al., 2001) or JUN-N-terminal kinase (JNK) (Strutt et al., 1997). Alternatively, DVL activates RAC to trigger JNK activity, which controls gene expression via JNK-dependent transcription factors (Fanto et al., 2000). The activation of WNT-PCP signaling results in cell polarity, cell migration and convergent extension [Convergent extension (CE) is a critical process by which the tissue of an embryo is restructured to converge (narrow) along one axis and extend (elongate) along a perpendicular axis by cellular movement] (Niehrs, 2012). The WNT-Ca^2+^ signaling pathway activates heterotrimeric G proteins to trigger phospholipase C (PLC) activity, which stimulates diacylglycerol (DAG) and inositol-1,4,5-triphosphate, type 3 (IP3) production leading to intracellular calcium flux (Slusarski et al., 1997), as well as calcineurin, calmodulin-dependent kinase II (CaMKII) and protein kinase C (PKC) activation (Kuhl et al., 2000; Wang et al., 2010). In addition, the transcriptional regulator nuclear factor associated with T cells (NFAT) is stimulated via the YES/CDC42/CK1α pathway and is translocated to the nucleus to control transcription of genes involved in morphogenesis, mesenchymal to epithelial transition and metastasis (Dejmek et al., 2006; Burn et al., 2011).

Herein, the review will focus on non-canonical WNT5B signaling and its role in both normal physiology and disease, especially in comparison to WNT5A.

## WNT5B Activity and Signaling

The WNT5 subfamily of WNT proteins consists of WNT5A and WNT5B. The Wnt5 gene is present in Cnidarians (diploblastic animals including freshwater polyps and hydroids, sea anemones and corals, and jellyfish) (Guder et al., 2006). Duplication can be observed in Xenopus, but not in all chordates, such as chickens. *WNT5B* is the genetic paralog of *WNT5A* (Bergstein et al., 1997). They have 87 and 80.5% amino acid identity in mice and humans, respectively (Gavin et al., 1990; Saitoh and Katoh, 2001). Both WNT5A and WNT5B show similar expression patterns and functions in some cell types, such as cardiomyocytes (Mazzotta et al., 2016; Albanese et al., 2017) and lung fibroblasts (van Dijk et al., 2016; Wu et al., 2019). However, WNT5A and WNT5B are expressed in non-overlapping patterns during mouse development (Gavin et al., 1990; Lickert et al., 2001; Church et al., 2002), suggesting that WNT5A and WNT5B activity are distinctly different during embryogenesis. Moreover, distinct effects of WNT5A and WNT5B are found in chondrocytes (Church et al., 2002; Yang et al., 2003), bone (Maeda et al., 2012; Brommage et al., 2014), adipocytes (Kanazawa et al., 2005; Maeda et al., 2012), mammary epithelial cells (Kessenbrock et al., 2017), and myeloid cells (de Rezende et al., 2020; see Table 1 and specific tissues below).

**TABLE 1:** Distinct effects of WNT5A and WNT5B.

**Organ/Tissue**	**Cell type**	**WNT5A**	**References**	**WNT5B**	**References**
Embryo		Controls convergent extension (z)	Ye et al., 2013	Controls convergent extension (z)	Ye et al., 2013
Palate		Knockouts had a short palate (z)	He et al., 2008	Knockouts had a short palate (z)	Rochard et al., 2016
Bone		*Wnt5a*^–/–^ showed skeletal defects in the mouse fetus (m) *Wnt5a*^±^ exhibited low bone mass (m)	Yamaguchi et al., 1999 Maeda et al., 2012	High-throughput *Wnt5b^–/–^* slightly increased bone mass (m)	Brommage et al., 2014
	Chondrocyte	Expressed at joint and perichondrium (c) Activated transition from proliferative chondrocyte zone to pre-hypertrophy zone by repressing *Ccnd1*, *Sox9*, and *Col2a1*, while increasing *p130* (m)	Church et al., 2002 Yang et al., 2003	Expressed at pre-hypertrophic zone (c) Promoted proliferative chondrocyte zone but suppressed the transition to pre-hypertrophy zone by elevating *Ccnd1*, *Sox9*, and *Col2a1*, while suppressed *p130* (m)	Church et al., 2002 Yang et al., 2003
	Osteoblast	Promoted osteoblastogenesis by upregulating *Lrp5/6* expression and activating β-catenin-dependent signaling pathway (m)	Okamoto et al., 2014	No report of the mechanism of action	
	Osteoclast	Increased osteoclastogenesis by upregulating RANK through ROR2/JNK signaling pathway (m)	Maeda et al., 2012	Activated osteoclast differentiation via RYK receptor (murine RAW264.7 monocytic cells)	Santiago et al., 2012
	Synovial mesenchymal stem cell	Aggravated joint degeneration by inducing senescence and inflammatory cytokines (h)	Huang et al., 2020	Promoted joint degeneration by inhibiting chondrocyte differentiation and ECM secretion (h)	Huang et al., 2020
Adipose tissue	Adipocyte	Suppressed adipogenesis (r)	Tang et al., 2018	Promoted adipogenesis and increased the level of *PPAR*γ, *C/EBP-α*, *ADIPOQ* and *LEP* (mouse 3T3-L1 cell line) Inhibited nuclear translocation of β-catenin (mouse 3T3-L1 cell line)	Kanazawa et al., 2004 Kanazawa et al., 2005
Pancreas	β-cell	Less expressed and colocalized with insulin- and glucagon-immunoreactive cells (m) No effect on NKX6.1 production in human iPSC (h)	Heller et al., 2002 Vethe et al., 2019	Strongly expressed and localized with insulin- and glucagon-immunoreactive cells (m) Increased the level of NKX6.1 in human iPSC (h)	Heller et al., 2002 Vethe et al., 2019
Cardiac tissue	Cardiomyocyte	No effect on pacemaker differentiation (z/h)	Ren et al., 2019	Induced pacemaker differentiation through activating β-catenin-dependent signaling pathway (z/h)	Ren et al., 2019
Nervous system	Differentiated neural stem cell	Slightly increased in differentiated cells (m)	Choi et al., 2011	Highly elevated in differentiated cells (m)	Choi et al., 2011
	Dorsal ganglia root and spinal cord dorsal horn	Decreased after morphine treatment and morphine withdrawal (m)	Wu et al., 2020	Strongly increased after morphine treatment and morphine withdrawal (m)	Wu et al., 2020
	Hippocampal dentate gyrus	Downregulated in ictal zone and peri-ictal zone after seizure (m)	Gupta and Schnell, 2019	Elevated in ictal zone but reduced in peri-ictal zone after seizure (m)	Gupta and Schnell, 2019
	Mechano-sensory Cell	No effect on cilia-mediated mechanosensory cells (z)	Louwette et al., 2012	Induced ciliogenesis in inner ear and migration of neuromast (z)	Louwette et al., 2012
Mammary gland	Mammary epithelial cell	Endogenously expressed and unable to transform C57MG cell morphology Increased mammosphere formation through RYK receptor (m) Inhibited mammary gland branching via ROR2 receptor (m)	Wong et al., 1994 Kessenbrock et al., 2017	Exogenously expressed and induced C57MG morphological transformation Suppressed mammosphere formation through ROR2 and RYK-independent receptors (m) No effect on mammary gland branching (m)	Wong et al., 1994 Kessenbrock et al., 2017
Lung	Alveolar epithelial progenitors (AEPs)	Represses the growth of lung organoid co-cultures of fibroblasts and epithelial progenitors (m) Highly expressed in COPD (h)	Wu et al., 2019 van Dijk et al., 2016	Represses the growth of lung organoid co-cultures of fibroblasts and epithelial progenitors (m) Represses the growth and differentiation of AEPs (m) Highly expressed in COPD (h)	Wu et al., 2019 van Dijk et al., 2016
Immune system	Myeloid cells	No effect on IL-3 and GM-CSF-induced myeloid differentiation (m) Knockdown *Wnt5a* did not affect thrombocyte production (z)	de Rezende et al., 2020 Louwette et al., 2012	Exhibited divergent effects on IL-3 and GM-CSF-induced myeloid differentiation (m) Knockdown *Wnt5b* showed severe thrombocytopenia (z)	de Rezende et al., 2020 Louwette et al., 2012
	T cells	Not expressed in thymocytes, peripheral T cells and epithelial cells (m)	Balciunaite et al., 2002	Expressed in thymocytes, peripheral T cells and epithelial cells (m) Highly expressed in CD4^+^ CD8^+^ double positive thymocytes and decreases by 75-95% during later stages of T cell development (m)	Balciunaite et al., 2002
	Whole blood	No change in expression in septic shock (m/h)	Gatica-Andrades et al., 2017	Increased expression in septic shock and correlated with IL-6, IL-10, and TNF (m/h)	Gatica-Andrades et al., 2017
	Lymphatic system	Increased migration of lymphatic endothelial cells, leading to elongated tube formation. (m)	Lutze et al., 2019	Necessary and sufficient for lymphatic endothelial specification (z/h) Enhances the lymph vessel formation, permeability and migration of lymphatic endothelial cell (h)	Nicenboim et al., 2015 Wang et al., 2017
Cancer	Breast cancer	Controversial: Correlated with better prognosis and induced tumorigenicity (h)	Zeng et al., 2016	Correlated with a worse prognosis in BLBC (h)	Yang et al., 2014; Jiang et al., 2019
	Colorectal cancer	Promotes migration and invasion (h) Inhibits cell proliferation and EMT (h)	Bakker et al., 2013 Cheng et al., 2014	Promoted cell proliferation, migration and invasion through the JNK signaling pathway (h)	Zhang et al., 2016
	Pancreatic cancer	Leads to pancreatic cancer progression and chemo-resistance (h)	Wei et al., 2014; Bo et al., 2016; Ram Makena et al., 2019	Not studied	
	Lung cancer	Overexpressed in NSCLC compared to normal tissue (h) Overexpression is correlated with poor overall survival (h)	Huang et al., 2005; Zhang et al., 2020	Overexpressed in NSCLC compared to normal tissue (h) Overexpression is correlated with poor overall survival (h)	Huang et al., 2005; Zhang et al., 2020
	Oral squamous cell carcinoma	TGF-β1 stimulated Slug which decreased WNT5A expression (h)	Hino et al., 2016	TGF-β1 stimulated Slug which increased WNT5B expression (h)	Hino et al., 2016
	Osteosarcoma	Correlated with advanced surgical stage and tumor metastasis (h) Increased migration and invasion of osteosarcoma cell lines (h)	Lu et al., 2012. Enomoto et al., 2009; Wang et al., 2018	Similarly expressed compared to the expression of *ROR2* (h) No study on the function in osteosarcoma	Morioka et al., 2009
	Ovarian cancer	Suppresses growth of epithelial ovarian cancer (h)	Bitler et al., 2011	Increases stemness (h)	Raghavan et al., 2019
	Brain cancer	Correlated with overall worse survival (h)	Xu et al., 2020	Correlated with better overall survival (h)	Xu et al., 2020
	Hepato-cellular carcinoma	No significant difference in WNT5A expression in tumor and adjacent-normal tissue (h)	Dong et al., 2019	High expression in tumor compared to adjacent-normal tissue (h)	Dong et al., 2019
	Leukemia	Highly expressed in CLL and correlates with CLL aggressiveness (h) Expression is lost in AML (h)	Janovska et al., 2016 Martin et al., 2010	Highly expressed in CLL and AML (h)	Janovska et al., 2016 Zheng et al., 2017

*z, zebrafish; c, chicken; m, mouse; r, rat; and h, human.*

*Wnt5b* was first identified in 1990 and was found to be expressed during embryogenic development (Gavin et al., 1990). Mouse *Wnt5b* is located on chromosome 6 (Church et al., 2009) and encodes a 3.2 kb single RNA species (Gavin et al., 1990) and in turn, 49 and 46.5 kDa proteins can be visualized by immunoblotting after post-translational modification (Smolich et al., 1993). Although mouse and human WNT5B show 87.74% gene similarity and 94% amino acid identity^1^, the rodent *Wnt5b* promoter and human *WNT5B* promoter are significantly divergent (Katoh and Katoh, 2005). Human *WNT5B*, located at chromosome 12p13.33 (Saitoh and Katoh, 2001), has 4 exons and alternative promotors that encode *WNT5B* into two isoforms differing at the first exon: isoform 1 (NM_032642.2) consists of exon 1A and isoform 2 (NM_030775.2) consists of exon 1B. Exons 1A and 1B correspond to the 5′-UTR, while exon 2 contains the starting methionine (Katoh and Katoh, 2005). The consequence of the differing exon usage is unknown.

WNT5B functions through the non-canonical WNT signaling pathway (Lin et al., 2010; Bradley and Drissi, 2011; Park et al., 2015; van Dijk et al., 2016; Mattes et al., 2018). While it is called β-catenin-independent signaling, WNT5B is often an antagonist to β-catenin signaling (Santiago et al., 2012; Mattes et al., 2018; Wu et al., 2019; Figure 1). The receptors and coreceptors [FZD protein(s)] to which WNT5B binds is still controversial. A structure-based prediction shows that the cystine-rich domain of FZD6 binds with WNT5B into a palm-shaped opening structure (Dahiya et al., 2019) and then activates a small G_0_ protein (Katanaev and Buestorf, 2009). In contrast, fluorescence recovery after photobleaching (FRAP) revealed that WNT5B did not affect FZD6 mobility, suggesting a lack of WNT5B-FZD6 interactions, at least in the HEK293T model system (Kilander et al., 2014). *In silico* analysis predicts that FZD8 has the highest affinity (Agostino et al., 2017; Dahiya et al., 2019), while *in vitro* and *in vivo* studies show that WNT5B can bind to FZD1 (Park et al., 2015), FZD2 (van Dijk et al., 2016), FZD4 (Mazzotta et al., 2016; Sarin et al., 2018), FZD5 (Sarin et al., 2018), FZD6 (Katanaev and Buestorf, 2009; Mazzotta et al., 2016), and FZD7 (Yin et al., 2020).

## Role of WNT5B in Development

WNT signaling is crucial for embryonic development in all animal species and the topic has been well-reviewed by others (van Amerongen and Nusse, 2009; Steinhart and Angers, 2018). The role of WNT5B signaling in embryonic development is less clear. In general, WNT signaling in embryonic development is context-dependent in both invertebrates and vertebrates. In embryos, WNT signaling has definitive roles for ventral-dorsal and anterior-posterior organization during various stages of early development. The organization of cells requires different WNT inputs to mediate planar cell polarity (PCP) during developmental processes such as gastrulation and neural tube closure. The polarization of node cells along the anterior–posterior axis of a mouse embryo was mostly unknown until the discovery of the asymmetric expression of *Wnt5a* and *Wnt5b* in the posterior side, which coincided with an anterior expression of WNT inhibitor SFRP (Minegishi et al., 2017). The opposing distribution of *Wnt5a*/*Wnt5b* gradients with that of SFRP guides intercellular signaling via PCP to polarize the cells along the anterior-posterior axis and to make a break of the left–right symmetry of the cells. The data suggest that WNT5A/5B have a redundant role in governing the break of left-right symmetry in cells, but it is unknown exactly which receptor/pathway this action is mediated through.

Vertebrate gastrulation is critical to establishing the germ layer and to coordinate the body axes, which are guided by cell movements. During gastrulation in zebrafish development, silencing of focal adhesion kinase (FAK1A) utilizing morpholinos led to the loss of convergent extension, impaired epiboly (cellular movement of squamous epithelium) and hypoblast cell migration. FAK1A combined with WNT5B rescued convergent extensions mediated by the FAK1A phenotype (Hung et al., 2020). Conversely, an injection of subthreshold levels of *Wnt5b* antisense morpholino oligonucleotides led to enhanced loss of convergent extension in the null FAK1A zebrafish. The results suggest that gradient increases of *Wnt5b* expression change or hamper or complement the loss of FAK1A activity on CE. Furthermore, CE was mediated by RAC1 and CDC42 actin dynamics. WNT5A also regulates convergent extensions in the zebrafish embryo (Ye et al., 2013).

Palate morphogenesis in the zebrafish at E4.5 is also formed by CE (Rochard et al., 2016). *Wnt5b* knockout zebrafish had a short palate. The ablation of *Wnt5b* affected the columnar organization but not the single layer stacking during CE. These results demonstrate that WNT5B controls the anterior-posterior axis in palate formation. WNT5A also regulates the anterior-posterior axis of the palate, similar to WNT5B (He et al., 2008). Interestingly, a translocation in the WNT5A locus has been implicated in human cleft palates (Blanton et al., 2004).

Cilia and basal bodies on the cell help control convergent extension. Basal body proteins [encoded by *bbs1*, *bbs4*, and *bbs6* (*mkks)*] were found to interact with WNT11 and WNT5B and signal through the WNT-PCP pathway (Gerdes et al., 2007). Knockout of BBS4 produced the strongest phenotype, as determined by defective tail extension. The authors suggest that WNT11 and WNT5B may have partially redundant roles, as mutants fail to extend axial tissues in zebrafish. Moreover, BBS4 was also shown to affect canonical WNT signaling in a mammalian cell system (using HEK293T cells) to measure ciliary function. Upon BBS4 suppression, β-CATENIN accumulated and increased nuclear Disheveled 3 (DVL3) localization, with increases in corresponding WNT reporter activity. Mechanistically, the authors link the stability of β-CATENIN protein degradation, in the absence of BBS4 expression, to BBS4’s ability to physically interact with the proteasomal subunit RPN10. These results demonstrate a link between WNT/β-catenin signaling and non-canonical signaling (WNT-PCP) in convergent extension.

## Role of WNT5B in Bone

WNT5B is involved in many aspects of bone physiology, including bone formation, bone maintenance and bone degradation. *Wnt5b* is expressed in mouse limb development; meanwhile, *Wnt5a* was not detected (Witte et al., 2009). In total long bone extracts, *Wnt5b* has the highest expression in young mouse bone (6 weeks old), decreases by adulthood (6 months old) and remains constant in aged mice (18 months old). *Wnt5a* shows the same pattern of expression, but its expression level is fourfold lower compared to *Wnt5b* at each timepoint (Rauner et al., 2008).

**FIGURE 2:**
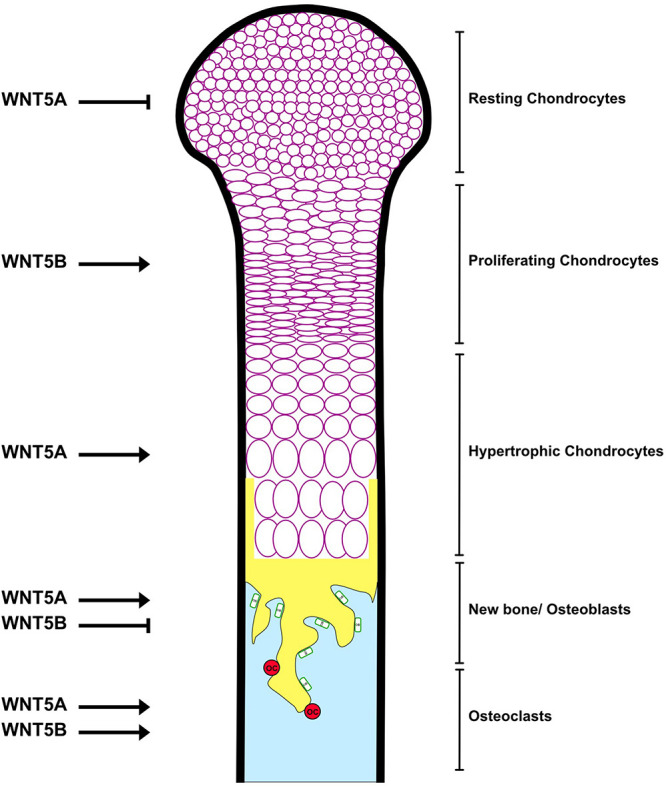
The roles of WNT5A and WNT5B in the bone. See the text for details.

WNT5B is expressed in mesenchymal stem cells (MSCs) (Fazzi et al., 2011; Charoenpanich et al., 2014) and the lineage cells, including chondrocytes (Church et al., 2002; Bradley and Drissi, 2011; Duesterdieck-Zellmer et al., 2015) and osteoblasts (Rauner et al., 2008). WNT5B is also expressed in osteoclasts (Santiago et al., 2012). WNT5A also has high expression in MSCs, as does WNT5B (Fazzi et al., 2011), but the level of WNT5A is higher than WNT5B in osteoblasts (Maeda et al., 2012; Kemp et al., 2014), adipose tissues (Akoumianakis et al., 2019), as well as osteoclast precursor bone marrow-derived macrophages (Maeda et al., 2012). High-throughput knockout of *Wnt5b* revealed an increase in bone mineral density (BMD) (Brommage et al., 2014), indicating that WNT5B affects bone negatively, but this has not been investigated beyond analyzing BMD. In comparison, knockout of *Wnt5a* results in skeletal defects in the mouse fetus and perinatal lethality (Yamaguchi et al., 1999). *Wnt5a*^±^ mice exhibit low bone mass with decreases in both osteoblastogenesis and osteoclastogenesis (Maeda et al., 2012), in contrast to the high bone mass in *Wnt5b* knockout mice. The skeletal phenotype of the WNT5A knockout mouse is also due to its proliferative and PCP effects in mesenchyme (Andre et al., 2015). The role of WNT5B in specific bone cells (chondrocytes, osteoblasts and osteoclasts) (Figure 2) and bone diseases (osteoarthritis and osteoporosis) is discussed below.

### Chondrocytes

Endochondral ossification is the major process for long bone formation in which mesenchymal cells must first condense and differentiate into chondrocytes to establish a cartilaginous structure before gradually being replaced by bone cells. During this process, chondrocyte regions can be divided into two zones, Zone I (articular and resting chondrocytes) and Zone II (proliferative chondrocytes), depending on cell morphologies, developmental functions and fates. Zone I corresponds to the epiphysis and contains two cell types: articular chondrocytes which will become permanent articular cartilage to maintain normal joint function throughout life and resting chondrocytes, which contain stem cell-like activity and embed into the growth plate (Abad et al., 2002). Meanwhile, Zone II maintains highly proliferative chondrocytes in the growth plate, which will undergo hypertrophy. The proliferative activity of Zone II is dependent on Zone I and requires tight regulation from systemic and local factors (Hunziker, 1994; Karp et al., 2000).

WNT5B is highly expressed in cartilage canal chondrocytes during neonatal development (Duesterdieck-Zellmer et al., 2015). In long bones, WNT5B is restricted to the pre-hypertrophic zone (Zone II) (Church et al., 2002), and acts as a local factor to activate proliferation of chondrocytes in Zone I by downregulation of *p130* (an Rb family transcription factor) and upregulation of *Ccnd1, Sox9, and Col2a1*, leading to the promotion of Zone II formation (Yang et al., 2003; Figure 2). Overexpression of *Wnt5b* delays terminal chondrocyte differentiation and hypertrophy by suppressing *Col10a1* expression, and results in an open skull, shortened long bones and reduced ossification in the embryo (Yang et al., 2003). Interestingly, misexpression of *Wnt5b* causes shortened limbs (Church et al., 2002), suggesting that precise conditions of WNT5B are important to regulate endochondral ossification.

WNT5B also controls other processes involved in cartilage development. WNT5B is required for craniofacial cartilage development in zebrafish. WNT5B regulates chondrocyte stacking in a Wnt/PCP-independent signaling pathway (Sisson et al., 2015) and increases chondrogenic cell proliferation via Wls and Fgf3 (Wu et al., 2015). WNT5B activates mesenchymal chondroprogenitor cell migration in a JNK-dependent manner (WNT-PCP signaling pathway). In addition, WNT5B decreases cell aggregation and prevents chondrocyte hypertrophy by reducing cadherin expression and destabilizing cadherin receptors via Src-mediated phosphorylation of membrane-bound β-catenin. Notably, phosphorylation of β-catenin by WNT5B results in decreased membrane-associated β-catenin and increased nuclear β-catenin, thereby activating β-catenin’s transcriptional activity (Bradley and Drissi, 2011), in contrast to inhibition of β-catenin signaling by WNT5B observed in osteoblasts, adipocytes, lung and other tissues (see below).

Although WNT5A and WNT5B both delay chondrocyte differentiation (Yang et al., 2003), their roles are distinct. WNT5A is expressed in joints and in the perichondrium, which is considered Zone I (Church et al., 2002). WNT5A inhibits proliferation and the transition from Zone I to Zone II by repressing *Ccnd1*, *Sox9* and *Col2a1* and elevating *p130*. Meanwhile, WNT5A promotes the transition from Zone II to the pre-hypertrophic zone, which will develop into the hypertrophic zone (Yang et al., 2003; Figure 2). Misexpression of *Wnt5a* results in truncated limbs and fused joints (Church et al., 2002), supporting the positive effect of WNT5A on hypertrophy and long bone formation.

### Osteoblasts

In osteoblast lineage cells, WNT5A and WNT5B exhibit redundant effects in mesodermal progenitor cells (MPCs), which have mesenchymal and endothelial differentiation potential (Petrini et al., 2009). WNT5A and WNT5B activate MPCs from quiescence and induce mesenchymal differentiation to MSCs through the FZD1/Calmodulin signaling pathway. The levels of WNT5A and WNT5B are high when the proliferation rate is low (early MSCs) and then decrease during exponential growth (late MSCs) (Fazzi et al., 2011). Then WNT5A and WNT5B expression increases during osteoblast differentiation (Hurson et al., 2007; Rauner et al., 2008; Okamoto et al., 2014), but the effects of WNT5A and WNT5B are divergent when MSCs commit to being osteoblasts. WNT5A promotes osteoblastogenesis by upregulation of *Lrp5/6* expression and results in enhanced β-catenin dependent signaling (Okamoto et al., 2014). Osteoblast lineage-specific *Wnt5a* conditional knockout mice show impaired osteoblastogenesis and decreased trabecular bone volume (Maeda et al., 2012). In contrast, there is some preliminary indication that WNT5B suppresses osteoblast differentiation (Figure 2). High expression of WNT5B, due to the regulation of circular RNA CDR1as, reduces β-catenin levels leading to decreased osteoblastogenesis in bone marrow mesenchymal stem cells (BMSCs) from steroid-induced osteonecrosis (Chen et al., 2020). However, the direct effects of WNT5B on normal osteoblast differentiation have not been studied.

Mechanical loading regulates bone remodeling by stimulating bone formation directly. Osteocytes, which contain sensors of strain energy, secrete sclerostin to inhibit WNT/β-catenin-dependent signaling, resulting in suppressed osteoblastogenesis in mechanical unloading conditions (Robling and Turner, 2009). The effects of mechanical loading on WNT5A and WNT5B were shown in osteoblast progenitors but have never been tested in osteocytes. Cyclic tension strain elevated WNT5A and WNT5B in BMSCs and MSCs from osteoporotic donors, respectively (Charoenpanich et al., 2014; Gu et al., 2018). In rat tendon-derived stem cells, uniaxial mechanical tension (UMT) activates osteoblast differentiation and upregulates WNT5A, WNT5B, ROR2 and RAC1, then WNT5A and WNT5B promote UMT-induced osteoblastogenesis via the activation of JNK (Liu X. et al., 2015). WNT5A also induces osteoblast differentiation from BMSCs through FZD4/JNK signaling under mechanical stimulation (Gu et al., 2018). Notably, the distinct actions of WNT5B, but not WNT5A, between normal and mechanical loading-induced osteoblast differentiation demonstrate that WNT5B can act differently in varied conditions.

Bone and teeth both have a mineralized extracellular matrix and have similar properties of differentiation. Ten WNT ligands are expressed in the dental epithelium and mesenchyme during mouse tooth development. *Wnt5b* is specifically expressed in the papilla mesenchyme of mouse incisors at E16 and E18 (Suomalainen and Thesleff, 2010). Mechanical forces (hydrostatic pressure) upregulate expression of *WNT5B* in human exfoliated deciduous teeth stem cells and activate odontoblast differentiation (Miyazaki et al., 2019), similar to what is observed after mechanical forces in rat tendon-derived stem cells (Liu X. et al., 2015).

Bone fracture healing requires several steps and signaling pathways to regenerate new bone (Marsell and Einhorn, 2011). Indirect suppression of WNT5B and β-catenin via miRNAs that target parathyroid hormone (Yao et al., 2018) and PANX3 (Jia and Zhou, 2018) results in inhibition of fracture healing. Moreover, knockout of Toll-like receptor 4 (TLR4), which inhibits the WNT/β-catenin signaling pathway, promotes fracture healing and increases β-catenin, WNT4, WNT5B, PCNA, and BMP-2 (Zhao et al., 2020), suggesting that β-catenin and WNT5B play similar roles in bone fracture healing. As both WNT/β-catenin-dependent and independent signaling pathways are involved in specific generative patterns of bone repair (Heilmann et al., 2013; Houschyar et al., 2018), WNT5B might regulate β-catenin activities to generate new bone. Nevertheless, the mechanisms that link WNT5B and β-catenin in this process are not understood.

### Osteoclasts

WNT5A and WNT5B are expressed in the osteoclast lineage and promote bone resorption by enhancing osteoclast differentiation (Figure 2). Nevertheless, their mechanisms of action are dissimilar. WNT5B increases TRAP activity through the RYK receptor and suppresses β-catenin signaling in osteoclasts (Santiago et al., 2012). Meanwhile, WNT5A is highly expressed, secreted from osteoblast lineage cells and increases osteoclastogenesis by upregulating receptor activator of nuclear factor-kB (RANK) in bone marrow macrophages (BMMs), which are osteoclast precursors, via the ROR2/JNK non-canonical signaling pathway (Maeda et al., 2012). Notably, the expression of the receptors RYK and ROR2 in osteoclast lineages differs between the Maeda et al. (2012) and Santiago et al. (2012) studies. RT-PCR shows that RYK is highly expressed in both undifferentiated and differentiated murine osteoclast precursor monocytic cells (RAW264.7), while ROR2 is less expressed, and knockdown of the RYK receptor abolishes the effect of WNT5B (Santiago et al., 2012), supporting the argument that WNT5B signals through RYK signaling. In contrast, Maeda et al. (2012) found that ROR2 is in BMMs and conditional knockout of *Ror2* in RANK-expressing cells prevents WNT5A-induced osteoclastogenesis. The two contrasting results may be due to using an *in vitro* vs. an *in vivo* system. Thus, further work will be required to determine the role of WNT5B in the differentiation and activity of osteoclasts.

### Bone Diseases

Osteoarthritis (OA) is a common age-dependent degenerative joint disease found in women more frequently than men. It is characterized by progressive degeneration of the components of extracellular matrix (ECM) of the articular cartilage and subchondral bone associated with secondary inflammatory factors (Dijkgraaf et al., 1995). Loss of the canonical WNT signaling pathway can be observed in OA pathogenesis. The specific knockout of β-catenin (*Ctnnb1)* at the superficial zone promotes OA development (Xuan et al., 2019) and β-catenin is decreased in OA, while p-JNK, p-CaMKII and p-PKC are increased (Huang et al., 2020). WNT5B is upregulated in OA patients (Hopwood et al., 2007; Shi and Ren, 2020) and has higher expression in trabecular bone from women compared to men (Hopwood et al., 2007), indicating that it is a candidate to stimulate OA progression. WNT5B activates synovial MSC (SMSC) proliferation and migration via the Hippo-YAP signaling pathway (Tao S.C. et al., 2017). However, WNT5B inhibits chondrocyte differentiation and ECM secretion as well as promotes fibrosis by increasing collagen type I secretion (Tao S.C. et al., 2017; Huang et al., 2020), leading to joint degeneration. For inflammatory-induced OA, lipopolysaccharide activates WNT5B expression in human chondrocyte cells (CHON-001 cells) (Shi and Ren, 2020). WNT5B expression correlates with TGF-β1, a key player in OA pathology, in osteoblasts from OA patients (Kumarasinghe et al., 2012). WNT5B also increased MMP13 expression in SMSCs, which may lead to joint degradation (Huang et al., 2020).

WNT5A is also elevated in OA patients (Martineau et al., 2017) and shows similar effects on SMSC proliferation and migration as WNT5B (Tao S.C. et al., 2017). Nevertheless, the inhibition of WNT5A on SMSC differentiation to chondrocytes is different from WNT5B. WNT5A aggravates joint degeneration by inducing senescence and inflammatory cytokines such as IL-1β and IL-6 in SMSCs, whereas WNT5B does not (Huang et al., 2020). Taken together, WNT5A and WNT5B are involved in OA pathogenesis but the mechanisms of action are different.

**FIGURE 3:**
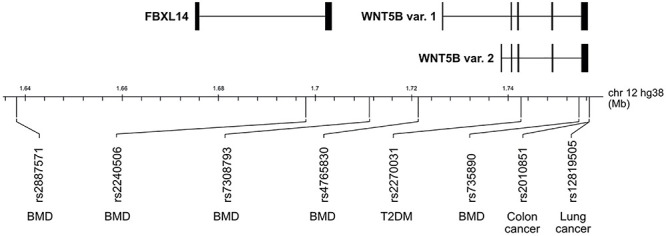
WNT5B single nucleotide polymorphisms (SNPs) in 12p13.33 implicated in disease. SNP rs2887571, rs2240506, rs7308793, rs4765830, and rs735890 are associated with variations in BMD. SNP rs2270031 is linked to Type 2 diabetes mellitus (T2DM). SNPs rs2010851 and rs12819505 are associated with colon cancer and lung cancer, respectively.

Osteoporosis is a bone metabolic disorder in which there is a loss of BMD due to an imbalance between bone formation and bone resorption, leading to an increased risk of bone fractures (Imel et al., 2014). The effect of WNT5B on osteoporosis is still unclear due to the lack of research on WNT5B in normal bone cells. However, several genetic studies link single nucleotide polymorphisms (SNPs) near WNT5B to osteoporosis. In 2012, a genome-wide meta-analysis first reported that WNT5B might affect BMD through SNP rs2887571 (Figure 3; Estrada et al., 2012). rs2887571 is correlated with BMD in children, and children carrying the risk allele at rs2887571 benefited from physical activity (Mitchell et al., 2016). However, since rs2887571 is located in a non-coding intergenic region, the true target gene or consequence of the polymorphism is still unknown. Other SNPs in the *WNT5B* gene (rs2240506, rs7308793, rs4765830, and rs735890) are also associated with BMD (Zheng et al., 2016). Interestingly, SNP rs735890 in WNT5B disrupts mRNA secondary structure and dictates the binding of miRNA, resulting in less translation of WNT5B and increased BMD (Amjadi-Moheb et al., 2018). The molecular mechanisms of how WNT5B is influenced by these SNPs should be further investigated.

## Role of WNT5B in Adipose Tissue

WNT5B has been shown to both transiently increase (Kanazawa et al., 2005) and decrease in early adipogenesis. However, both studies demonstrated that WNT5B activates adipocyte differentiation by elevating the level of adipocyte markers, such as peroxisome proliferator-activated receptor-γ (PPARγ), C/EBPα, AP2, Adiponectin and Leptin (Kanazawa et al., 2004; van Tienen et al., 2009). Furthermore, WNT5B abolishes the effect of WNT3A-suppressed adipogenesis (Kanazawa et al., 2005) by inhibiting β-catenin nuclear translocation and suppressing canonical WNT targeted genes such as *IGF-1*, *WISP-1*, and *VEGF-C* (Kanazawa et al., 2005; van Tienen et al., 2009). Conversely, WNT5A inhibits adipogenesis. *Wnt5a*^–/–^ mice have an increased number of adipocytes (Maeda et al., 2012) and WNT5A suppresses adipocyte differentiation from rat adipose-derived stem cells *in vitro* (Tang et al., 2018). This demonstrates the opposite effects of WNT5A and WNT5B in adipocytes, as well as in chondrocytes and osteoblasts, all of which originate from MSCs.

## Role of WNT5B in the Pancreas

WNT5B and WNT5A are expressed in embryonic mesenchyme where β-cells differentiate during mouse pancreatic development. WNT5B is strongly upregulated and localizes around insulin- and glucagon-immunoreactive cells; WNT5A, in contrast, has less expression and low localization with these cells (Heller et al., 2002). WNT5A increases ROR2 on the cell membrane, while WNT5B elevates the level of c-JUN, the downstream signaling of JNK (Vethe et al., 2019), suggesting that WNT5A and WNT5B act through the β-catenin-independent signaling pathway in pancreatic β-cells. In this study, the receptor for WNT5B was not analyzed, nor was the receptor for WNT5A, beyond the observation of increased ROR2 on the cell membrane. WNT5A and WNT5B together were shown to increase differentiation of β-like cells from induced pluripotent stem cells. In addition, WNT5A together with WNT5B or WNT5B alone, but not WNT5A alone, increase the level of NKX6.1, a transcription factor that controls β-cells fate, identity and function (Schaffer et al., 2013; Taylor et al., 2013), in human induced pluripotent stem cells differentiated to β-like cells (Vethe et al., 2019).

## Role of Wnt5B in Type 2 Diabetes Mellitus

A case-control association study showed that SNP rs2270031, located in a WNT5B intron (Figure 3), is strongly associated with type 2 diabetes mellitus (T2DM) in a Japanese population (Kanazawa et al., 2004). In addition, a study in a UK population combined rs2270031 with rs7903146 (located in an intron of *TCF7L2*, a transcription factor for *glucagon-like peptide-1)*, to show a risk effect for T2DM (Salpea et al., 2009). The necessity to combine the two genes to show an effect might be due to the fact that Caucasian subjects rarely have the risk allele at rs2270031. WNT5B regulates both adipocytes and pancreatic cells, two main cell types affected in diabetes, and mis-regulation of WNT5B via this SNP or otherwise in these cell types may lead to disease. Therefore, the effects of WNT5B in T2DM need further investigation.

## Role of WNT5B in Cardiac Tissue

WNT5A and WNT5B are expressed at various stages of heart development. They direct the commitment of mesoderm to cardiogenic mesoderm by elevating *MESP1*, the earliest cardiogenic marker, via the ROR2/JNK WNT signaling pathway (Mazzotta et al., 2016). WNT5A and WNT5B also stimulate the differentiation of cardiomyocytes via FZD4/6/Ca^2+^ WNT signaling pathway in late development (Mazzotta et al., 2016). However, only WNT5B promotes pacemaker cardiomyocytes, which are essential for the initiation and maintenance of proper heart rhythm through the activation of the canonical β-catenin/WNT signaling pathway (Ren et al., 2019). Interestingly, *activation* of the canonical pathway by WNT5B is observed here, as it is in chondrocytes, but in contrast to *inhibition* of β-catenin signaling by WNT5B observed in osteoblasts, adipocytes, lung and other tissues.

Aortic valve calcification causes aortic valve stenosis, which induces heart failure. WNT5A and WNT5B show the same effects on calcification development. Their expression is significantly, positively correlated with pathological calcified valves. WNT5A and WNT5B reduce proliferation and induce apoptosis of human aortic valve interstitial cells, increase osteogenic gene expression and increase calcium deposition and formation in calcified nodules *via* the JNK/Akt1/MAPK38 signaling pathway (Albanese et al., 2017). Fetuin A, a calcification inhibitor, reduces the expression of *WNT5A* and *WNT5B* (Khan et al., 2020).

## Role of WNT5B in the Nervous System

WNT5B is moderately expressed in the human and zebrafish fetal brain, especially in midbrain-hindbrain boundary constriction in zebrafish and is required for basal constriction via activation of focal adhesion kinase (FAK) (Gutzman et al., 2018). Then its expression decreases in adulthood (Saitoh and Katoh, 2001) similar to WNT5A (Gavin et al., 1990). The effects of WNT5A and WNT5B are both different and redundant in the nervous system, depending on cell types and contexts. Neural stem cells (NSCs) have the capacity to differentiate into either neurons, astrocytes or oligodendrocytes in the central nervous system (CNS). *Wnt5b* is increased more than twofold, while *Wnt5a* is only slightly elevated, in differentiated cells compared to NSCs (Choi et al., 2011). The median motor column (MMC) contains a set of motor neurons arranged in a longitudinal array and innervates axial muscles. WNT5B and WNT5A redundantly control MMC identity and connectivity by establishing a ventral^*high*^ to dorsal^*low*^ signaling gradient, subsequently promoting the persistent expression of LIM Homeobox protein 3 and 4, the transcription factors for cell specification (Agalliu et al., 2009).

WNT5B is also required for the mechanosensory system in mammalian and lower vertebrates. In zebrafish (*Danio rerio*), WNT5B regulates ciliogenesis in the inner ear and migration of neuromasts to the lateral line, a sensory organ of fishes, through the RGS18-mediated WNT-Ca^2+^ signaling pathway, whereas WNT5A does not have any effect on cilia-mediated mechanosensory cells (Louwette et al., 2012). In the juvenile rat cochlea, WNT5B is expressed in non-sensory supporting cells, and more strongly expressed in fibrocytes of the spiral limbus and spiral ligament (Daudet et al., 2002). This suggests a function of WNT5B in late cochlea differentiation, as well as auditory function, although no functional experiments have been performed to date.

WNT5B and WNT5A have the same effects on the development and degradation of retinal and optic nerves. Both proteins regulate retinal neuropil formation in the outer retina. WNT5A and WNT5B are selectively secreted by rod bipolar cells to rod photoreceptors, and then control the patterning of the outer plexiform layer via the RYK/FZD4/FZD5/DVL signaling pathway (Sarin et al., 2018). WNT5A and WNT5B are also elevated in a mouse model of retinal degradation (Yi et al., 2007), suggesting that they function during photoreceptor injury. However, the mechanism of action requires further investigation. Moreover, WNT5A and WNT5B are expressed in the neural retina, but not in retinal pigment epithelium (RPE) in the mouse embryo (Liu et al., 2003). A study in human embryonic stem cell-derived RPE cells supported that WNT5B and its downstream signaling have to be suppressed, while high expression of BMP7 is required in order to successfully go through epithelialization of the RPE cells (Choudhary et al., 2015).

WNT5B also controls optic nerve regeneration in lower vertebrates. Axon growth in zebrafish requires the activation of small G-proteins, such as CDC42 and Rac1, whereas RhoA has to be suppressed. *Wnt5b* is increased, while *Wnt10a* is decreased and *Wnt5a* has no change, after optic nerve transection. WNT5B stimulates the CDC42/Rac1/JNK/PAK signaling pathway and represses RhoA via CDC42, together with the reduction of β-catenin level in the nucleus due to the decrease of WNT10A. Both of these actions lead to axon extension (Matsukawa et al., 2018).

The expression of WNT5B is affected in several nerve injuries and neuropathology. Morphine treatment induces WNT5B accumulation in the dorsal ganglia root (DRG) and it is exported to the central terminus of the spinal cord dorsal horn (DH) after morphine withdrawal. Translocated WNT5B at the DH then increases Ca^2+^ influx via RYK, resulting in behavioral and neurochemical alteration of morphine withdrawal. Meanwhile, the level of *Wnt5a* expression is decreased after morphine treatment and morphine withdrawal (Wu et al., 2020). Chronic constriction injury-induced neuropathic pain and complete Freund’s adjuvant-induced chronic inflammation also elevate the level of *Wnt5b* expression in the DRG. Notably, the expression level of *Ryk* is raised in the same pattern as *Wnt5b* and a RYK antagonist abolishes the effects of the WNT/RYK/CaMKII/NR2B signaling pathway in neurons (Liu S. et al., 2015; Wu et al., 2020). However, there is no direct investigation of the effect of WNT5B on nerve injury. In addition, epilepsy is neural network remodeling in the hippocampus dentate gyrus, which associates with the alteration of WNT signaling pathways. WNT5B is significantly dysregulated between the ictal zone and peri-ictal zone, the center and remodeling network region after seizure induction, respectively. In contrast, *Wnt5a* is downregulated in both regions (Gupta and Schnell, 2019). Nevertheless, the mechanisms of action of WNT5B in epilepsy are poorly understood. Lastly, an epigenome-wide association study revealed that alteration in DNA methylation at the first intron of the longest isoform of *WNT5B* is associated with Alzheimer’s disease progression (Smith et al., 2019). However, the consequences of the methylation and different isoform functions remain unknown.

Together, these data suggest that WNT5B has varied expression in both the CNS and the peripheral nervous system, and exhibits several roles during embryonic development and pathogenesis, such as in neuron identity and patterning, neuron regeneration, and the mechanosensory system.

## Role of WNT5B in the Mammary Gland

*Wnt5a* and *Wnt5b* are expressed in the more differentiated luminal epithelial cells of the mammary gland (Ji et al., 2011). *Wnt5b* is also expressed in the mammary gland during mid-pregnancy and disappears in lactation, while *Wnt5a* is expressed in early pregnancy (Gavin and McMahon, 1992). In the mammary gland WNT5B signals through ROR2 and RYK-independent receptors. WNT5B inhibits mammosphere formation by downregulating proliferation genes, such as *Mcm2* and *Ki67*, as well as luminal differentiation genes, such as *Gata3.* WNT5A, in contrast, increases the number and size of mammospheres via the RYK receptor, and suppresses mammary gland branching via the ROR2 receptor (Kessenbrock et al., 2017), whereas WNT5B does not have any effect on mammary gland branching. These data, again, suggest distinct roles of WNT5A and WNT5B. Further work on the role of WNT5B in the mammary gland should be performed, as there are numerous reports of WNT5B in breast cancer (see below).

## Role of WNT5B in the Lung

Both WNT5A and WNT5B are expressed in the lung and have been implicated in lung pathogenesis, including COPD and lung cancer. WNT5A and WNT5B are secreted from lung fibroblasts in a paracrine manner to inhibit alveolar epithelial progenitors, while inducing epithelial differentiation. WNT5B, but not WNT5A, induces expression of Aquaporin 5 (Aqp5, a marker for alveolar type I cells) and Surfactant protein C (Sftpc, a marker for alveolar epithelial type II cells). WNT5B also shows a stronger repressive effect on alveolar organoid formation compared to WNT5A. However, canonical WNT/β-catenin signaling is repressed by both WNT5A and WNT5B in these cells. WNT5A and WNT5B seem to have similar functions, but are not identical (Wu et al., 2019).

Chronic obstructive pulmonary disease (COPD) is caused by defective epithelial lung repair associated with an abnormal inflammatory response. Alteration of fibroblast functions also plays an important role in COPD pathogenesis. WNT5B has higher expression in COPD patients (Heijink et al., 2016), suggesting a role in its pathogenesis. Furthermore, TGF-β1, a key player in fibroblast activation to repair lung injury, increases *WNT5B* expression and then WNT5B stimulates ECM production (fibronectin and α-smooth-muscle actin) and myofibroblast differentiation via FZD8 (Spanjer et al., 2016). WNT5B induces an inflammatory response in MRC-5 human fibroblasts and primary human lung fibroblasts by increasing IL-6 and CXCL8 secretion via the FZD2/Tak1/JNK signaling pathway (van Dijk et al., 2016). WNT5A is also highly expressed and impairs lung repair in COPD patients through the WNT/β-catenin-independent signaling pathway (Baarsma et al., 2017). These studies indicate that non-canonical WNT5A and WNT5B have similar roles in the lung and activating the development of COPD.

## Role of WNT5B in Hematopoiesis

Hematopoietic stem cells (HSCs) generate the balance between lymphoid and myeloid cell types which impact immunity efficiency and tissue homeostasis. WNT5B is expressed in HSCs (de Rezende et al., 2020) and has been reported in the development of myeloid and lymphoid lineage cells.

Acceleration of myelopoiesis in the elderly contributes to the development of chronic diseases associated with aging. Primitive cells such as HSCs and multipotent progenitors are more sensitive to IL-3, which supports stem cell maintenance, while committed myeloid progenitors are more sensitive to Granulocyte-Macrophage-Colony Stimulating Factor (GM-CSF), which drives differentiation and proliferation. WNT5B exhibits divergent effects on IL-3 and GM-CSF-induced myeloid differentiation, which relate to aging-associated myeloid imbalance. WNT5B enhances HSCs and progenitors by suppressing IL-3-mediated myeloid differentiation and non-canonical WNT signaling genes such as *c-Fos*, *c-Jun*, and *Cdc42*. Meanwhile, WNT5B accelerates GM-CSF-driven myelopoiesis and upregulates non-canonical WNT signaling genes, leading to progenitor cell exhaustion. Notably, osteoclasts are derived from the monocyte/macrophage lineage and WNT5B may play a role in osteoclast progenitor cells to affect bone mass. Furthermore, WNT5B controls megakaryocyte differentiation through RGS18 as depletion of *Wnt5b* in zebrafish embryo shows severe thrombopenia (Louwette et al., 2012). Interestingly, *Wnt5a* is expressed in primitive HSCs and niche cells, as is *Wnt5b*, but WNT5A does not affect IL-3- and GM-CSF-induced HSC differentiation (de Rezende et al., 2020).

The primary lymphoid organ for T-cell development is the thymus. The thymus functions to ensure that T-cells are selected to prevent autoimmunity. The T-cell section for non-autoimmune T-cells is mediated by both distinct thymic stromal cells and cortical and medullary epithelial cells (Anderson and Jenkinson, 2001). The full maturation of thymic epithelial cells (TECs) occurs in the third pharyngeal pouch (ppIII) of the thymus. Growth and development of TECs (Anderson and Jenkinson, 2001) are dependent on Forkhead Box N1 (FoxN1) expression, and in its absence, epithelial morphogenesis in the thymus is aberrant. These epithelial cells lack the ability to attract lymphoid precursors to the thymus primordium. *Wnt5b* expression is found in thymocytes, peripheral T cells and epithelial cells. Meanwhile, *Wnt5a* has no expression in these cells. Interestingly, *Wnt5b* is highly expressed in CD4^+^ CD8^+^ double-positive thymocytes and decreases by 75–95% during later stages of T cell development. Moreover, the transduction of cortical TEC 1-2 cells with *Wnt5b* upregulates FoxN1 and WNT5B is strictly localized with FoxN1 in ppIII. This suggests that WNT5B is involved in lymphocyte differentiation by regulating FoxN1 (Balciunaite et al., 2002), but the mechanism of WNT5B on T cells is still poorly understood. In addition, WNT5B also contributes to a dysregulated immune response and the production of inflammatory cytokines to the infection. WNT5B, but not WNT5A, increases in whole blood from patients with septic shock and directly correlates with IL-6, IL-10, and TNF (Gatica-Andrades et al., 2017).

## Role of WNT5B in the Lymphatic System

The lymphatic system origins have had two opposing models for close to 100 years, one suggests a venous origin for lymphatic endothelial cells, and the other a more random coalescence of discontinuous, independent lymphatic vessels arising from mesenchymal-derived cells. A study in zebrafish demonstrated that lymphatic progenitor cells, which arise from a specialized angioblast within the cardinal vein that can also give rise to arterial veins, are governed by WNT5B activity (Nicenboim et al., 2015). WNT5B is necessary and sufficient for lymphatic endothelial specification. WNT5B promoted the angioblast to lymphatic transition in both zebrafish and human embryonic stem cells, suggesting this function by WNT5B is evolutionarily conserved. WNT5B has also been shown to be secreted from tumor cells to induce tumor lymphangiogenesis (Wang et al., 2017). WNT5B enhanced the lymph vessel formation, permeability and migration of lymphatic endothelial cells. These findings can potentially lead to the development of regeneration of lymphatic vessels for human usage or inhibition of lymphangiogenesis in cancer.

WNT5A also increased migration of lymphatic endothelial cells, leading to elongated tube formation. This was demonstrated in mice, using the dermal lymphatic vascular system, and not compared to WNT5B in the same system (Lutze et al., 2019).

## Role of WNT5B in Cancer

### Breast Cancer

WNT1 was originally identified due to its ability to transform mammary gland cells. WNT2, WNT3A, WNT7A, WNT7B, WNT10B, and WNT5B are also highly transforming of mouse mammary cells. In contrast, WNT5A (and WNT4 and WNT6) failed to induce transformation (Wong et al., 1994).

*WNT5B* is overexpressed in the majority of Triple Negative (TNBC)/Basal-Like Breast Cancer (BLBC) cell lines and primary patient samples and is correlated with a worse prognosis (Yang et al., 2014; Jiang et al., 2019). In addition, WNT5B was found to be amplified in 3.2% of breast cancers, although the type of breast cancer with WNT5B amplification was not described (Jiang et al., 2019). Knockdown of *WNT5B* inhibited the proliferation, migration and invasion of Bcap-37 and MDA-MB-231 cells *in vitro* and reduced the expression of basal-like markers (Yang et al., 2014; Jiang et al., 2019). Mechanistically, WNT5B was shown to promote TAZ activation by promoting the transcription of SLUG, which is a master regulator of epithelial-mesenchymal transition (EMT) as well as a regulator of TAZ nuclear localization and subsequent activation (Samanta et al., 2018). In addition, knockdown of *WNT5B* in MDA-MB-231 and Bcap-37 cells showed reduced tumor growth in BALB/c-nu mice compared with control cells. Targeting WNT5B (and all WNTs) through the addition of WNT inhibitors pyrvinium pamoate (a CK1α activator) or LGK-974 (a Porcupine inhibitor) also reduced tumor growth and proliferation *in vivo* (Jiang et al., 2019).

The regulation of WNT5B expression in breast cancer (or other cell types) is not well-studied. However, it was shown that WNT5B is suppressed by miR145-5p in three basal-like breast cancer cell lines. Mechanistically, insulin-like growth factor-2 mRNA-binding protein 3 (IMP3), which is correlated with TNBC, stabilizes *WNT5B* by suppressing miR145-5p.

WNT5A and WNT5B also have different roles in mammary gland development and TNBC. In contrast to WNT5B, many publications show WNT5A as a tumor suppressor in breast cancer, correlating its loss with a worse prognosis (Zeng et al., 2016). However, the opposite has also been shown: WNT5A is higher in breast carcinomas and induces tumorigenicity in breast cancer (Iozzo et al., 1995; Zeng et al., 2016). Therefore, the roles of WNT5A and WNT5B in breast cancer need further research.

### Pancreatic Cancer

Genomic analysis of pancreatic ductal carcinoma revealed four major subtypes (Bailey et al., 2016) and the WNT signaling pathway was found to be enriched in the squamous subtype, which is the most aggressive subtype. Specifically, overexpression of canonical WNT ligands such as *WNT2* and *WNT7A* has been implicated in pancreatic progression via persistent activation of β-catenin signaling. In addition, the non-canonical WNT ligand WNT5A has been shown to lead to pancreatic cancer progression and chemoresistance (Wei et al., 2014; Bo et al., 2016; Ram Makena et al., 2019).

Only a few studies have suggested roles for WNT5B in normal or cancerous pancreatic cells. Although WNT5B has not been studied in pancreatic cancer patient samples, *WNT5B* mRNA is highly expressed in the pancreatic cancer cell line PANC-1 (Harada et al., 2017). Panc-1 cancer cells (and the lung adenocarcinoma cell line A549) can induce the invasive ability of neighboring epithelial cells both *in vitro and in vivo*. WNT3 and WNT5B are secreted from mesenchymal-transitioned cancer cells to induce the metastatic potential of neighboring epithelial cells (Kato et al., 2014). Knockout of *WNT5B* in Panc-1 cells reduced migration and invasion, but not proliferation. In control PANC-1 cells, addition of TGF-β increased the expression of *Snail* and *Vimentin* mRNA and decreased *E-cadherin*. However, in *WNT5B* knockout cells, TGF-β-dependent increases of *Snail* and *Vimentin* mRNA were reduced and the expression of *E-cadherin* mRNA decreased. These results suggest that WNT5B may be involved in TGF-β-induced EMT (Harada et al., 2017).

WNT5B has also been shown to be secreted in exosomes from PANC-1 cells. WNT5B-associated exosomes promoted cancer progression in a paracrine manner, as seen in their ability to induce proliferation and migration on A549 lung cancer cells (Harada et al., 2017). The contribution of WNT5B in exosomes vs. secretion needs to be explored in both pancreatic cancer and other cell types.

### Lung Cancer

Many WNT ligands, both canonical and non-canonical, and WNT signaling proteins are misregulated in lung cancer. Specifically, both WNT5A and WNT5B are overexpressed in non-small cell lung cancers (NSCLC) compared to normal tissue and their overexpression is correlated with poor overall survival (Huang et al., 2005; Zhang et al., 2020). Knockdown of *WNT5B* reduced the proliferation and growth rate of lung adenocarcinoma both *in vitro* and *in vivo* by inducing cell-cycle arrest at the G1/S phase. Mechanistically, WNT5B was shown to regulate the metabolism of the lung cancer cells, including the amino acid transporter LAT1. In addition, miR-5587-3p was shown to repress WNT5B in these cells (Zhang et al., 2020).

In a group of non-small cell lung carcinomas, variants for rs12819505 (Figure 3) were significantly associated with worse prognosis (Stewart et al., 2014). The function of this SNP, which is a few hundred base pairs downstream of the WNT5B coding sequence is unknown.

### Colorectal Cancer

WNT signaling is highly important in colorectal cancer (CRC) through the regulation of proliferation, differentiation, and cell fate. WNT signaling is activated in normal intestinal crypts, which is crucial for stem cell maintenance and tissue homeostasis. The canonical WNT/β-catenin pathway is constitutively activated in more than 90% of colorectal cancers. APC suppresses β-catenin-mediated WNT signaling and is thus a tumor suppressor gene. Germline mutations in APC lead to familial adenomatous polyposis (FAP) and account for about 1–2% of CRC. Somatic mutations in APC have also been found in a majority of sporadic CRC (Aoki and Taketo, 2007).

In contrast to β-catenin-dependent signaling, very little is known about WNT5B in CRC. At least five CRC cell lines express WNT5B and the addition of WNT5B to COLO 205 cells promoted cell proliferation, migration and invasion through the JNK signaling pathway (Zhang et al., 2016). Examination of patient samples for mRNA and/or protein levels could determine if there is a role for WNT5B in CRC.

The polymorphism rs2010851 in the 3′ UTR of *WNT5B* (Figure 3) was shown to predict tumor recurrence in stage II colon cancer. This specific *WNT5B* SNP is significant because it shows for the first time that a WNT polymorphism can predict early tumor recurrence in colorectal cancer (Paez et al., 2014), and suggests that more research needs to be done to understand the consequences of both the SNP and WNT5B in CRC.

Similarly, the role of WNT5A in CRC is not clear, as WNT5A has both been shown to promote migration and invasion (Bakker et al., 2013) and to inhibit cell proliferation and EMT (Cheng et al., 2014). WNT5A is down-regulated in a majority of colon cancers and suppressed during colon cancer metastasis (Li and Chen, 2012; Tao J. et al., 2017). The contradictory roles of WNT5A in CRC could be due to two different *WNT5A* splice forms, with each form having opposing functions (Huang et al., 2017).

### Oral Squamous Cell Carcinoma

Expression profiles from 40 patients with oral squamous cell carcinoma (OSCC) revealed a small (1.5-fold) increase in *WNT5B* mRNA in 40% of cases. In addition, WNT5B mRNA and protein were higher in patients with nodal metastases (Wang et al., 2017). As in many other cancer types, knockdown of WNT5B decreased OSCC cell line proliferation and migration (Takeshita et al., 2014; Wang et al., 2017). WNT5B was further shown to activate CDC42 and RhoA, which are involved in cell migration (Takeshita et al., 2014). In the HSC-4 OSCC cell line, TGF-β1 stimulated Slug which increased WNT5B expression, while decreasing WNT5A expression. In turn, WNT5B increased the expression of MMP-10, a matrix metalloproteinase that induces tumor progression and invasion (Hino et al., 2016).

### Osteosarcoma

Osteosarcoma is a cancer that starts in the bone, and affects mostly children, especially during puberty. In osteosarcoma, it has been identified that the interaction of WNT5B and ROR2 can enhance cell migration, which supports a potential role of ROR2 and WNT5B in the metastatic pathway of osteosarcoma cells. *ROR2* was overexpressed in 56% of patient samples and the expression pattern of *WNT5B* expression is concurrent with that for *ROR2*. Knockdown of *ROR2* suppressed proliferation and invasion of osteosarcoma cells, suggesting that ROR2 and WNT5B could be promising therapeutic targets for osteosarcoma patients (Morioka et al., 2009).

While only one paper exists on the significance of *WNT5B* in osteosarcoma, several highlight the importance of *WNT5A*. WNT5A was overexpressed in 81% of 42 osteosarcoma immunohistochemistry samples and was significantly correlated with advanced surgical stage and tumor metastasis (Lu et al., 2012). Specifically, WNT5A increased migration and invasion of osteosarcoma cell lines (Enomoto et al., 2009; Wang et al., 2018). As in CRC, the different isoforms of WNT5A have different roles in osteosarcoma cell lines (Vaidya et al., 2016). WNT5B also has two different promoters and splice forms, suggesting the need to also investigate WNT5B isoforms in osteosarcoma.

### Ovarian Cancer

WNT5B is important in ovarian cancer, particularly in ovarian cancer stem cells (OCSC). Immune cells, including macrophages, are enriched in ovarian cancer ascites. Raghavan et al. (2019) demonstrated that WNT5B from macrophages induces expression of the stem cell marker *ALDH* in the OCSC in a paracrine manner. They also showed a 50% decrease in β-catenin protein expression after WNT5B knockdown, indicating cross-talk with canonical WNT signaling (Raghavan et al., 2019). Zong et al. (2020) also demonstrated the importance of WNT5B in OCSC and demonstrated that WNT5B induces ALDH expression in OCSC. Their research proposed that the tumor suppressor Disabled homolog 2-interacting protein (DAP2IB) down-regulates WNT5B in OCSC and proposed the pathway in which EZH2 inhibits DAP2IB, which inhibits WNT5B signaling. Addition of recombinant WNT5B to the ovarian cancer cell line OVCAR3 induced JNK and c-Jun phosphorylation and downstream stemness related genes (Zong et al., 2020). They further suggested that combining an EZH2 inhibitor, or other epigenetic therapy, with a non-canonical WNT pathway inhibitor for ovarian cancer would be optimal.

While not much is known about the clinical implications of *WNT5B*, high expression of *WNT5A* has been significantly correlated with ovarian cancer stage, poorer overall survival and poorer progression-free survival (Peng et al., 2011). WNT5A has been shown to regulate ovarian cancer invasion and migration. In addition, it has been shown that WNT5A may mediate vasculogenic mimicry and EMT in ovarian cancer cells via PKC-α signaling (Qi et al., 2014). In contrast, Bitler et al. (2011) propose that loss of *WNT5A* expression predicts worse outcomes in patients with Epithelial Ovarian Cancer (EOC) and that *WNT5A* expression suppresses the growth of EOC cells by initiating cellular senescence. While both WNT5B and WNT5A have been shown to be expressed in ovarian cancer, their functions are distinct.

### Brain Cancer

There are more than 120 types of brain and central nervous system tumors. The presence and/or biology of WNT5B in these tumors are mostly unknown. The WNT5B locus has been shown to be amplified in astrocytomas (Schiffman et al., 2010), but this has not been further described. The expression of *WNT5B* has been shown in gliomas and atypical teratoid rhabdoid tumors (ATRT), but studies beyond gene expression have not been performed. *WNT5B* expression was correlated with better overall survival, in contrast to patients with elevated *WNT5A* expression that had overall worse survival (Xu et al., 2020). In a set of ATRT samples, *WNT5B* was upregulated in 19 of the 20 samples. Furthermore, WNT inhibitors were shown to decrease proliferation in these cells. WNT5B was shown to bind to FZD1 but the functional role of WNT5B in ATRT patients is still unknown (Chakravadhanula et al., 2015).

### Hepatocellular Carcinoma

Hepatocellular carcinoma (HCC) can be divided into well-differentiated (epithelial, conserved hepatocyte morphology) and poorly differentiated (mesenchymal, more motile and invasive) tumors. A study of HCC cell lines revealed that canonical WNT ligands (WNT3, WNT8B, and WNT10B) are expressed in well-differentiated tumors, while non-canonical WNT ligands such as WNT5B and WNT5A are correlated with poorly differentiated tumors. This study further proposes a potential crosstalk mechanism by which non-canonical WNT5A inhibits canonical WNT signaling (Yuzugullu et al., 2009).

The expression of WNT ligands was also evaluated in 360 HCC tumor tissues and 50 adjacent non-tumor tissues by RNA-sequencing. The expression of five WNT genes (*WNT2B*, *WNT3A*, *WNT6*, *WNT8B*, and *WNT10B*) was higher in tumor tissues than adjacent normal cells. Six WNTs, including WNT5B, showed the opposite pattern of expression, suggesting that WNT5B may have anti-tumor activity in HCC. Meanwhile, there was no significant difference in WNT5A expression in tumor and adjacent normal tissue (Dong et al., 2019).

### Leukemias

Several groups have shown that WNT5B is significantly higher in chronic lymphocytic leukemia (CLL) than normal B cells. Specifically, WNT5B is higher in patients without IgV mutations (Lu et al., 2004; Memarian et al., 2009; Janovska et al., 2016). This higher expression of WNT5B correlates with CLL aggressiveness in patients. WNT5A also is highly expressed in CLL and correlates with CLL aggressiveness. However, the signaling induced by WNT5A and WNT5B is not identical, as only WNT5A induced DVL3 phosphorylation and only WNT5A correlated with basal migration (Janovska et al., 2016).

WNT5B was also shown to be highly expressed in EpCAM^+^ cells (leukemia stem cells) in acute myeloid leukemia (AML) and to associate with the resistance of EpCAM^*high*^ myeloid leukemia cells to cytotoxic chemotherapy (Zheng et al., 2017). In contrast, WNT5A expression is epigenetically silenced in a majority of patients with AML (Martin et al., 2010).

## Conclusion

WNT5B is often assumed to have a similar function as WNT5A because they belong to the WNT5 subfamily and share a high amino acid identity. They have similar roles in convergent extension during development, lymphatic development, MMC identity in the nervous system, retinal development, and aortic valve calcification (see Table 1). However, WNT5B exhibits unique, often opposing, effects on development and homeostasis in many tissue types including bone, adipose tissue, mammary gland, and myeloid cells. Non-canonical WNT5B functions through both WNT-PCP and WNT-Ca^2+^ signaling pathways, which are responsible for not only cell movement, but also cell proliferation, and cell differentiation. Disrupted WNT5B signaling leads to the progression of diseases such as osteoarthritis, osteoporosis, obesity, type 2 diabetes mellitus and chronic diseases associated with aging, as well as cancers. Understanding the mechanistic effects of WNT5B, which are not well known, could be translated to the development of potential therapies.

## Author Contributions

All authors listed have made a substantial, direct and intellectual contribution to the work, and approved it for publication.

## Conflict of Interest

The authors declare that the research was conducted in the absence of any commercial or financial relationships that could be construed as a potential conflict of interest.

## Footnotes

1 www.genecards.org
