# Corrigendum: WNT5B in Physiology and Disease

**DOI:** 10.3389/fcell.2021.724948

**Published:** 2021-07-23

**Authors:** Sarocha Suthon, Rachel S. Perkins, Vitezslav Bryja, Gustavo A. Miranda-Carboni, Susan A. Krum

**Affiliations:** ^1^Department of Orthopaedic Surgery and Biomedical Engineering, University of Tennessee Health Science Center, Memphis, TN, United States; ^2^Department of Experimental Biology, Faculty of Science, Masaryk University, Brno, Czechia; ^3^Department of Cytokinetics, Institute of Biophysics, Czech Academy of Sciences, Brno, Czechia; ^4^Division of Hematology and Oncology, Department of Medicine, University of Tennessee Health Science Center, Memphis, TN, United States; ^5^Center for Cancer Research, University of Tennessee Health Science Center, Memphis, TN, United States

**Keywords:** Wnt5B, Wnt signaling, development, cancer, Wnt5a

In the original article, there were two errors.

(1) Our information on Wnt modification and secretion was out of date. Mouse Wnts are not palmitoleated on cysteines—that was an error in mutational analysis by Karl Willert. All the cysteines in Wnt are engaged in disulfide bonds (DOI 10.1126/science.1222879, 10.1074/jbc.m114.575027). However, a new reference describes WNT palmitoylation in zebrafish WNT3A (Dhasmana et al., 2021).

(2) The WLS protein binds to Wnt in the ER, not the Golgi. The Golgi localization of WLS was also an error due to the use of an epitope tag on the c-terminus (10.1016/j.devcel.2014.03.016, 10.1016/j.cell.2020.11.038).

A correction has been made to ***the introduction, paragraph number*** 2

The WNT family now contains 19 WNT genes, falling into 12 WNT subfamilies in mammalian genomes. All WNT genes encode proteins around 40 kDa in size and contain highly conserved cysteines (Miller, 2002; Clevers and Nusse, 2012). Mammalian WNT proteins are palmitoylated at conserved serine residues by a special palmitoyl transferase, Porcupine (PORCN), in the endoplasmic reticulum (Takada et al., 2006; Galli et al., 2007; Rios-Esteves et al., 2014). Zebrafish WNT3 is lipidated at both cysteine and serine residues (Dhasmana et al., 2021). The activity of PORCN is essential for the secretion of WNT ligands. Then, the seven-transmembrane protein Wntless/Evi (Wls) in the endoplasmic reticulum escorts mature hydrophobic WNT proteins to be secreted at the plasma membrane or released in exosomes, leading to both autocrine and paracrine effects (Banziger et al., 2006; Routledge and Scholpp, 2019).

Accordingly, the following reference has been added to the original article:

Dhasmana, D., Veerapathiran, S., Azbazdar, Y., Nelanuthala, A. V. S., Teh, C., Ozhan, G., et al. (2021). Wnt3 is lipidated at conserved cysteine and serine residues in zebrafish neural tissue. *Front. Cell Dev. Biol*. 9:671218. 10.3389/fcell.2021.671218

And the following reference has been removed from the original article:

Willert, K., Brown, J. D., Danenberg, E., Duncan, A. W., Weissman, I. L., Reya, T., et al. (2003). Wnt proteins are lipid-modified and can act as stem cell growth factors. *Nature* 423, 448–452.

The authors apologize for these errors and state that this does not change the scientific conclusions of the article in any way. The original article has5 been updated.

## Publisher's Note

All claims expressed in this article are solely those of the authors and do not necessarily represent those of their affiliated organizations, or those of the publisher, the editors and the reviewers. Any product that may be evaluated in this article, or claim that may be made by its manufacturer, is not guaranteed or endorsed by the publisher.
